# Scenarios of future mpox outbreaks among men who have sex with men: a modelling study based on cross-sectional seroprevalence data from the Netherlands, 2022

**DOI:** 10.2807/1560-7917.ES.2024.29.17.2300532

**Published:** 2024-04-25

**Authors:** Marc C Shamier, Luca M Zaeck, Hannelore M Götz, Bruno Vieyra, Babs E Verstrepen, Koen Wijnans, Matthijs RA Welkers, Elske Hoornenborg, Brigitte AGL van Cleef, Martin E van Royen, Kai J Jonas, Marion PG Koopmans, Rory D de Vries, David AMC van de Vijver, Corine H GeurtsvanKessel

**Affiliations:** 1 Department of Viroscience, Erasmus University Medical Center, Rotterdam, the Netherlands; 2 Department of Public Health, Municipal Public Health Service Rotterdam-Rijnmond, Rotterdam, the Netherlands; 3 Department of Public Health, Erasmus University Medical Center, Rotterdam, the Netherlands; 4 Department of Infectious Diseases, Public Health Service Amsterdam, Amsterdam, the Netherlands; 5 Amsterdam UMC location AMC, University of Amsterdam, Department of Medical Microbiology and Infection Prevention, Amsterdam, the Netherlands; 6 Department of Pathology, Erasmus University Medical Center, Rotterdam, the Netherlands; 7 Faculty of Psychology and Neuroscience, Maastricht University, Maastricht, the Netherlands

**Keywords:** mpox, monkeypox virus, serosurveillance, transmission model, outbreak preparedness, antibodies

## Abstract

**Background:**

Following the 2022–2023 mpox outbreak, crucial knowledge gaps exist regarding orthopoxvirus-specific immunity in risk groups and its impact on future outbreaks.

**Aim:**

We combined cross-sectional seroprevalence studies in two cities in the Netherlands with mathematical modelling to evaluate scenarios of future mpox outbreaks among men who have sex with men (MSM).

**Methods:**

Serum samples were obtained from 1,065 MSM attending Centres for Sexual Health (CSH) in Rotterdam or Amsterdam following the peak of the Dutch mpox outbreak and the introduction of vaccination. For MSM visiting the Rotterdam CSH, sera were linked to epidemiological and vaccination data. An in-house developed ELISA was used to detect vaccinia virus (VACV)-specific IgG. These observations were combined with published data on serial interval and vaccine effectiveness to inform a stochastic transmission model that estimates the risk of future mpox outbreaks.

**Results:**

The seroprevalence of VACV-specific antibodies was 45.4% and 47.1% in Rotterdam and Amsterdam, respectively. Transmission modelling showed that the impact of risk group vaccination on the original outbreak was likely small. However, assuming different scenarios, the number of mpox cases in a future outbreak would be markedly reduced because of vaccination. Simultaneously, the current level of immunity alone may not prevent future outbreaks. Maintaining a short time-to-diagnosis is a key component of any strategy to prevent new outbreaks.

**Conclusion:**

Our findings indicate a reduced likelihood of large future mpox outbreaks among MSM in the Netherlands under current conditions, but emphasise the importance of maintaining population immunity, diagnostic capacities and disease awareness.

**Key public health message**

**What did you want to address in this study and why?**

In this study we aimed to identify how immunity against mpox in a specific population will affect the risk and size of a future outbreak. To this end, we identified the proportion of individuals with poxvirus-specific antibodies among men who have sex with men (MSM) in the Netherlands following vaccination or infection. These data were used to establish a stochastic model that estimates the risk of future mpox outbreaks.

**What have we learnt from this study?**

We identified detectable antibody levels in just under half of samples from MSM who frequented the Centres for Sexual Health in Rotterdam and Amsterdam (the Netherlands). Using a mathematical model, we demonstrated that the 2022 vaccination campaign, while only having a negligible effect on the Dutch 2022-23 outbreak itself, would result in a distinct reduction of the size and duration of future outbreaks. However, additional measures are needed to completely prevent these.

**What are the implications of your findings for public health?**

These findings underscore the importance of establishing and maintaining monkeypox virus-specific immunity in the at-risk population through vaccination, while emphasising the necessity of a multifaceted approach, including sustained disease awareness and upkeep of diagnostic capacities.

## Introduction

The orthopoxvirus genus includes several viruses that can infect humans including (i) the now eradicated variola virus, which caused smallpox; (ii) vaccinia virus (VACV), which was used as an early smallpox vaccine; and (iii) monkeypox virus (MPXV), the causative agent of mpox. A VACV-based vaccine was employed as part of the global immunisation campaign to eradicate smallpox, but routine smallpox vaccination was discontinued globally in the 1970s following the successful elimination of smallpox. As a consequence, population susceptibility to orthopoxviruses has gradually increased [1]. This growing pool of susceptible individuals is thought to have directly contributed to the recent global mpox outbreak with over 90,000 reported cases predominantly among men who have sex with men (MSM) [2,3]. The Dutch 2022–23 outbreak consisted of over 1,250 reported cases and peaked in July 2022. Of the first 1,000 cases, 99% were males with a median age of 37 years, of whom 95% identified as MSM [4].

Prior to the 2022–23 mpox outbreak, studies conducted in different regions of the world demonstrated significant variations in orthopoxvirus seroprevalence levels. Orthopoxvirus seroprevalence in blood donors was shown to be less than 10% in non-endemic countries i.e. countries without an animal reservoir of mpox such as France, the Lao People’s Democratic Republic and Bolivia [5]. In contrast, seroprevalence levels of 51% and 60% were measured in endemic countries with an mpox animal reservoir such as Côte d’Ivoire and the Democratic Republic of the Congo, respectively [6]. Although different methodologies were used that limit direct comparisons of seroprevalence rates, these findings underscore a high susceptibility on a population level for MPXV infections in non-endemic countries before the 2022–23 outbreak.

A third-generation smallpox vaccine based on the replication-deficient poxvirus modified vaccinia virus Ankara (MVA) (MVA-BN, also known as Imvanex, JYNNEOS or Imvamune) was rapidly employed in vaccination campaigns across different countries during the 2022–23 mpox outbreak to interrupt MPXV transmission in high-risk populations, replacing previously employed second-generation smallpox vaccines (replication-competent vaccinia viruses) [7-10]. We have previously demonstrated that, while a two-dose MVA-BN immunisation series in non-primed individuals induced a cross-reactive immune response against MPXV, levels of MPXV-neutralising antibodies were comparatively low [11]. Simultaneously, we demonstrated the presence of neutralising antibodies in individuals who likely received childhood smallpox vaccination over 70 years post vaccination [11]. This confirmed previous assumptions that vaccinia virus-based vaccination results in the induction of neutralising antibodies cross-reactive against MPXV in humans [12], which has since been corroborated by other groups [13,14]. While a specific correlate of protection for mpox has not yet been determined, research involving non-human primates demonstrated that vaccine efficacy of Dryvax, a first-generation vaccine, is affected by B-cell but not T-cell depletion. Furthermore, passive immunisation with human vaccinia-neutralising antibodies conferred protection against severe disease in unvaccinated macaques [15]. These findings underscore the significance of humoral immunity in conferring protection against mpox, at least in the non-human primate model. Recent studies from Israel [7], the United Kingdom (UK) [8] and the United States (US) [9] reported a vaccine effectiveness for MVA-BN against mpox of between 36% and 86%, which was comparable to that of the first-generation smallpox vaccine of 58–85% [4,16,17]. Recently, breakthrough infections in previously vaccinated individuals and re-infections in individuals who had already contracted mpox have been reported [10,18-22], raising concerns about the longevity of immune responses and the effectiveness of orthopoxvirus-specific immune responses in preventing novel outbreaks.

In contrast to previous outbreaks of mpox in non-endemic areas [23,24], the 2022–23 outbreak exhibited several distinct epidemiological characteristics [3,25]. These included its unprecedented scale, the occurrence of disease mainly among MSM and sexual contact as a primary mode of transmission [26]. A modelling study based on the UK outbreak highlighted a substantially higher basic reproduction number (R_0_) within the MSM sexual network compared with non-sexual household transmissions [26]. The deceleration of the outbreak in the second half of 2022 was attributed to the lack of susceptible individuals, either due to vaccination- or infection-induced immune responses, combined with increased awareness and behavioural changes particularly within the context of sexual interactions [27-29]. Despite recognising the importance of population immunity for the prevention of future outbreaks, none of these studies performed immunological assessments and uncertainties persist regarding the current level of immunity among the at-risk population.

To estimate the impact of population immunity on the size and duration of potential future mpox outbreaks, we assessed the seroprevalence of VACV-specific antibodies among 1,065 MSM in the two largest cities in the Netherlands after the peak of the 2022–23 mpox outbreak. The study population comprises MSM presenting at Centres for Sexual Health (CSH), who likely exhibit higher levels of sexual activity than the general Dutch MSM population. Consequently, they are more likely to have been invited for vaccination and/or to have been exposed to the virus, therefore representing the group at highest risk of MPXV infection. The observed seroprevalence levels in combination with published data on vaccine effectiveness [7-9,16] and serial interval, defined as the period between the onset of symptoms in a primary case and the onset of symptoms in a secondary case [30], were subsequently used in a stochastic transmission model to estimate the magnitude of future mpox outbreaks.

## Methods

### Study population

Centres for Sexual Health offer testing for sexually transmitted infections (STI) to those at high risk such as MSM. In the Netherlands, mpox testing at CSH was introduced during the early phase of the outbreak in 2022 and mpox vaccination began to be offered mid-July 2022 through the public health service. We analysed residual serum samples obtained from MSM by the CSH in Rotterdam (n = 315) and Amsterdam (n = 750). Sera were collected in September 2022 after the mpox outbreak had peaked in the Netherlands and vaccination had been introduced. Vaccination was offered by CSH to MSM clients who were (current or prospective) HIV pre-exposure prophylaxis (PrEP) users, were HIV infected or were at high risk for STI. The latter was defined as notified for STI exposure, having been diagnosed with an STI recently or having a history of multiple sexual partners. Individuals born before 1974 (cessation of smallpox vaccination for the general population in the Netherlands) were inferred to have received childhood smallpox vaccination.

### Detection of vaccinia virus -specific IgG antibodies

For the detection of VACV-specific IgG antibodies, an in-house screening ELISA was employed using a VACV Elstree-infected HeLa cell lysate as antigen as described previously [11]. We have previously demonstrated a positive correlation between the presence of VACV-specific binding antibodies and MPXV-neutralising as well as MPXV-binding antibodies [11]. Absorbance was measured at 450 nm using an Anthos 2001 microplate reader and corrected for absorbance at 620 nm. Values of optical density (OD) measured at a wavelength of 450 nm (OD_450_ values) were obtained with mock-infected cell lysates and subtracted from the OD_450_ value obtained with the VACV-infected cell lysates to determine a net OD_450_ response. A positive control based on a pool of two sera from post-MVA-BN individuals who had also received childhood smallpox vaccination was included on every ELISA plate. The ELISA was validated using a set of 85 sera from orthopoxvirus-naïve individuals (expected negative for VACV-specific antibodies), and a set of 57 sera from double-dose MVA-BN-vaccinated individuals collected 28 days after the second dose (expected positive for VACV-specific antibodies). For validation results see Supplementary Figure S1. For the estimation of seroprevalence levels, a cut-off optical density measured at a wavelength of 450 nm (OD_450_) of 0.2 was used, corresponding to a sensitivity of 100%, and a specificity of 89.9%. This cut-off was chosen over a cut-off with slightly higher specificity to allow for the detection of low OD_450_ values in the early stages of infection or shortly after vaccination. A borderline area was identified up to an OD_450_ of 0.35, and the borderline-positive sera are displayed visually distinct from the positive samples in the figures.

### Stochastic model

A mathematical stochastic model was used to model mpox transmission. The model was developed in MATLAB and is available on GitHub (www.github.com/dvandevijver/mpox_model). The model was calibrated to the cumulative number of individuals diagnosed with mpox in the 2022–23 outbreak in the Netherlands, the seroprevalence at the end of the outbreak and the number of vaccinated individuals. Details are displayed in the Supplementary Figure S2, Supplementary Table S1 and Supplementary Methods. In the model, seroprevalence was represented as the sum of individuals (i) who had been vaccinated (childhood vaccination or newly vaccinated in 2022); (ii) infected at the end of the runs; or (iii) recovered from MPXV infection. The model was seeded by 1–10 individuals who were initially infected with MPXV following an event with a high number of potential exposures. The model stratified individuals who were not infected based on vaccination status (not vaccinated, historically smallpox vaccinated before 1974 or recently MVA-BN vaccinated during the 2022–23 outbreak). Using literature estimates, vaccination was assumed to reduce the risk of infection by 85% (range 75–95%) in historically vaccinated individuals [17] and by 78% (95% confidence interval (CI): 54–89%) in recently vaccinated individuals [7-9]. Upon infection, individuals first enter an exposed state in which they are not infectious to others. Individuals become infectious after a serial interval of 8 days (95% CI: 6.5 – 9.9 days) [30]. We assume individuals remain infectious until diagnosed (within 1 to 21 days after symptom onset) [31], after which they will end high risk behaviour and consequently will not transmit MPXV to others. At the beginning of the outbreak in 2022, there was no awareness of mpox and diagnostic tests were not available. The outbreak in the Netherlands started between the middle of April and the middle of May 2022. After 23 May, we assumed that awareness had increased, resulting in reduced time between symptom onset and diagnosis and a reduction in new sexual partners by up to 50% [32,33]. Individuals who had recovered from mpox were assumed not to be infectious.

We defined 'demographic turnover' as the annual rate at which older individuals transition out of the sexually active Dutch MSM population due to factors such as ageing or reduced sexual activity and are replaced by younger individuals. This turnover reflects the shift in population immunity as the exiting group is more likely to have acquired immunity through vaccination or past infections. Given the absence of detailed demographic data specific to the MSM community's sexual activity patterns, we extrapolated our assumptions from the closest available statistic, which is the national crude birth rate. In the Netherlands, this rate declined from 2.08% in 1960 to 0.95% in 2022 [34]. Consequently, for the purpose of our model, we adopted a hypothetical turnover rate ranging from 1% to 5% per year to simulate a spectrum of potential demographic changes affecting the size of mpox outbreaks, while keeping the total population size constant.

### Statistical analysis

A chi-squared test for equality of two proportions was used to compare the seroprevalence percentage between Amsterdam and Rotterdam. For continuous variables, medians and interquartile ranges (IQR) were reported if data was not normally distributed, and 95% confidence intervals (95% CI) were provided to indicate the precision of the estimates. Data were visualised using Prism (v10.0; GraphPad).

## Results

### Study population

Characteristics of MSM who visited CSH in Rotterdam and Amsterdam in September 2022 are summarised in the Table. Of the 315 MSM visiting the CSH in Rotterdam, median age was 34 years (IQR: 28–42) and 13.6% (43/315) of the men were born before 1974 and likely received a childhood first-generation smallpox vaccination. Most of the participants were invited for MVA-BN vaccination due to (current or prospective) PrEP usage (59.4%, 187/315), high-risk behaviour (8.3%, 26/315) or HIV infection (1%, 3/315). At the time of this cross-sectional study, 19.7% (62/315) of the participants had received one dose of the MVA-BN vaccine with a median time between sampling and vaccination of 26 days and 14.6% (46/315) had received two doses with a median time between sampling and last dose of 9 days. Among those who visited the Centre for Sexual Health in Rotterdam, five individuals (1.6%, 5/315) had tested positive for mpox since May 2022.

**Table:** Characteristics of men who have sex with men visiting the Centres of Sexual Health in Rotterdam and Amsterdam, September 2022 (n = 1,065)

Characteristics	Rotterdam (n = 315)		Amsterdam (n = 750)	
	n	%	n	%
Age (years)				
< 20	3	1.0	12	1.6
20–29	101	32.1	286	38.1
30–39	112	35.6	258	34.4
40–49	56	17.8	102	13.6
50–59	28	8.9	66	8.8
60–69	13	4.1	24	3.2
70–79	2	0.6	2	0.3
Vaccination type				
MVA-BN only	97	30.8	NA	
MVA-BN and historic smallpox vaccination^a^	28	8.9	NA	
Historic smallpox vaccination^a^	15	4.8	NA	
Unvaccinated	175	55.6	NA	
Reason MVA-BN vaccination offered				
Not invited	99	31.4	NA	
PrEP use or waitlist	187	59.4	NA	
PLWH	3	1.0	NA	
Recent STI exposure or treatment, or multiple sexual partners	26	8.3	NA	
MVA-BN vaccination (n = 125)				
1 dose	79	25.1	NA	
Days since last dose, median (IQR)	27 (19.0–34.75)		NA	
2 doses	46	14.6	NA	
Days since last dose, median (IQR)	9 (7.0–13.5)		NA	
PCR-confirmed infections				
Infected	5	1.6%	NA	

IQR: interquartile range; MVA-BN: modified vaccinia Ankara–Bavarian Nordic; NA: not available; PLWH: people living with HIV; PrEP: (HIV) pre-exposure prophylaxis.

^a^ Historic smallpox vaccination was inferred for those born before cessation of childhood smallpox vaccination in the Netherlands in 1974.

Of the 750 MSM visiting the Centre for Sexual Health in Amsterdam, median age was 32 years (IQR: 27–40), and 13.3% (100/750) were born before 1974 and presumably received childhood smallpox vaccination. Data on MVA-BN vaccination and MPXV infection were not available for the Amsterdam cohort.

### Seroprevalence of vaccinia virus-specific antibodies

**Figure 1:**
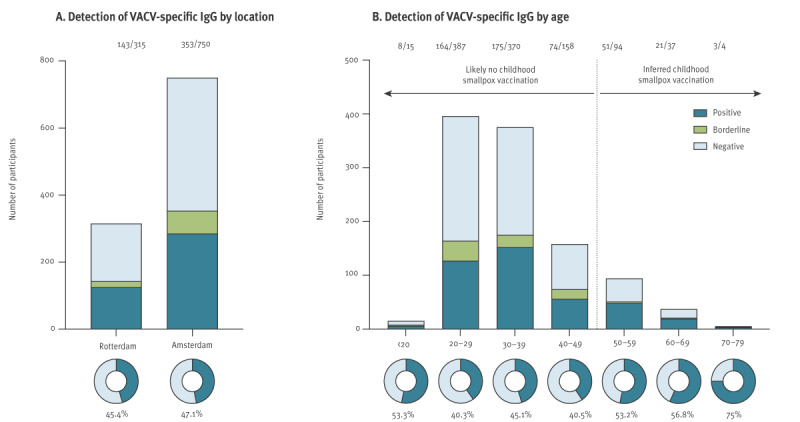
Seroprevalence of vaccinia virus-specific antibodies among men who have sex with men, Rotterdam (n = 315) and Amsterdam (n = 750), 2022

Vaccinia virus-specific IgG antibodies were detected in 143 of 315 sera (45.4%) from MSM in Rotterdam (Figure 1 and Supplementary Figure S3). Of these positive sera, 18 of 143 (12.6% of positives or 5.7% of total) were borderline positive (OD_450_ between 0.2 and 0.35). Vaccinia virus-specific IgG antibodies were detected in 353 of 750 (47.1%) sera from MSM in Amsterdam. Of these, 68 of 353 (19.3% of positives or 9.1% of total) were borderline positive (Figure 1A). Seroprevalence was lowest in 20–29-year-olds, which comprised most MSM, and highest in the oldest group of 70–79 years (Figure 1B). In all groups above 50 years of age, who have likely been historically vaccinated against smallpox, VACV-specific antibodies were detected in at least 50% of individuals. Overall, the seroprevalence of VACV-specific antibodies among MSM was comparable between Rotterdam and Amsterdam (χ^2^ = 0.257, p = 0.61).

OD_450_: optical density measured at a wavelength of 450 nm; VACV: vaccinia virus.

Detection of VACV-specific IgG in n = 1,065 serum samples from MSM visiting the Centres for Sexual Health in Rotterdam (n = 315) and Amsterdam (n = 750) using an in-house screening ELISA grouped by location (A) or age (B). Samples were considered positive with an OD_450_>0.35 (dark blue), borderline-positive with an OD_450_ between 0.35 and 0.2 (green), and negative with an OD_450_<0.2 (light blue). Seroprevalence levels were estimated based on the less stringent cut-off of OD_450_>0.2, including borderline-positive samples, and are shown as donut graphs. The numbers above the plots indicate the number of seropositive participants among the respective population.

### Fitting of the stochastic monkeypox virus transmission model

**Figure 2:**
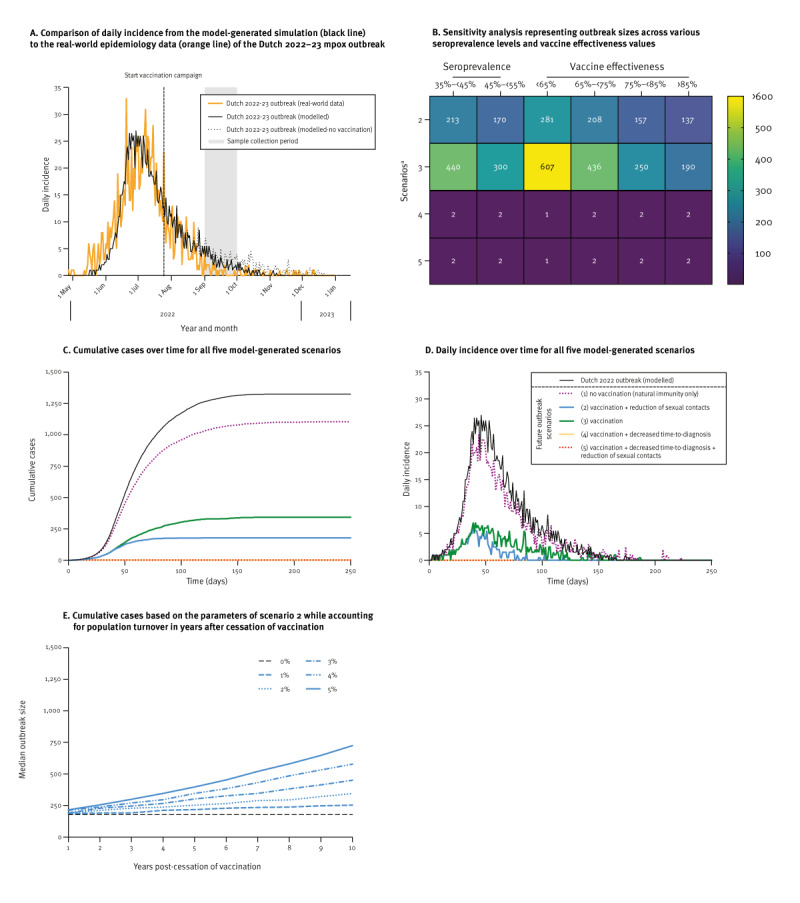
Monkeypox virus transmission model among men who have sex with men (MSM) in the Netherlands

A stochastic MPXV transmission model was generated to estimate the risk of a future mpox outbreak among MSM using our serological observations and available literature data on MPXV serial interval [30] and vaccine effectiveness [7-9,16]. This model was calibrated to parameters derived from the Dutch 2022–23 mpox outbreak. The model-generated simulation (black line) demonstrated comparability of daily incidence to real-world data (orange line) on the Dutch 2022–23 mpox outbreak from the Dutch National Institute for Public Health and the Environment (RIVM) [35] (Figure 2A). Simulation of the 2022–23 Dutch mpox outbreak using our model yielded a median total cumulative case count of 1,325 (IQR: 1,262–1,419) over a duration of ca 25 weeks. The number of actual reported cases in the Netherlands during the same period was 1,259. Simulating the 2022-2023 Dutch mpox outbreak in the absence of a vaccination campaign indicated that risk group vaccination only led to a marginal decrease of cumulative cases (1,427, IQR: 1,321–1,565, dotted black line [modelled – no vaccination] vs. 1,325, IQR: 1,262–1,419, solid black line [modelled]).

The stochastic transmission model uses the seroprevalence data reported here (range between 35% and 55%; Figure 1) in combination with available literature data on serial interval and vaccine effectiveness and was calibrated to parameters derived from the Dutch 2022–23 mpox outbreak.

^a^ Five different scenarios were modelled: (1) simulation assuming that no vaccination campaign had occurred in 2022 and only individuals with prior infections or childhood vaccinations would be (partially) protected against infection; (2) a partially vaccinated population (seroprevalence 35–55%) with a reduction of sexual partners within the risk group comparable to the original outbreak; (3) same as scenario 2 but without a reduction of sexual contacts; (4) a partially vaccinated population (seroprevalence 35–55%) with a decreased time-to-diagnosis comparable to the end of the outbreak; (5) same as scenario 4 but with a reduction of sexual contacts (Supplementary Methods).

In Panel A the peak of the curve from real-world epidemiology data was overlayed with the peak of the model-generated curve.

In Panel E the population turnover was simulated with variable turnover rates between 1% and 5%; 0% represents no turnover and hence maintenance of initial seroprevalence levels. Total population size remained unchanged.

### The effect of vaccination and early diagnosis on future outbreak size

A sensitivity analysis was conducted to examine the influence of distinct seroprevalence levels and a varied range of vaccine effectiveness on the stochastic model (Figure 2B). To investigate the impact of immunity conferred by prior infections and vaccinations on a potential future mpox outbreak we conducted several simulations (Figure 2C, 2D and Supplementary Table S2). First, we simulated a future outbreak assuming that no vaccination campaign had occurred in 2022 and only individuals with prior infections or childhood vaccinations would be (partially) protected against infection (scenario 1). In this situation a new outbreak would only be slightly reduced in size compared with the 2022–23 outbreak, with a cumulative case count of 1,105 (IQR: 1,000–1,206).

Subsequently, we modelled the impact of MVA-BN vaccination in 2022 by considering the range of seroprevalence levels between 35% and 55% based on the data reported here (scenario 2). In both this scenario and the next, we assumed similar diagnostic capacities as those in place before the 2022–23 outbreak. We observed a large reduction in outbreak size, with a median of 179 cases (IQR: 108–265), an average daily incidence of 1.5 cases and a duration of approximately 17 weeks. This marks an 86.4% reduction in outbreak size compared with our model’s reproduction of the 2022–23 Dutch outbreak. In this simulation, similar to the 2022–23 outbreak, we assume that the population at risk reduced their number of sexual partners by up to 50%.

We separately included a simulation in a vaccinated population, without any change in the number of sexual partners (scenario 3). In this scenario, the total outbreak size was 344 cases (IQR: 167–526), with an average daily incidence of 1.8 cases.

Next, we examined the influence of a reduced time-to-diagnosis. At the beginning of the 2022–23 outbreak, there was a diagnostic delay resulting in a delay in case isolation. The duration of infectiousness decreased during the later stages due to reduced time-to-diagnosis, resulting in an earlier isolation on average. In a simulation involving a partially vaccinated population with a time-to-diagnosis comparable to the later stages of the 2022–23 outbreak (scenario 4), no outbreak occurred (median case count of 2, IQR: 0–6). In this situation, a reduction in risk behaviour did not have any additional impact (scenario 5). Even with lower vaccine effectiveness and seroprevalence ranges, the combination of vaccination and sufficient laboratory testing capacity indicated to be effective in preventing outbreaks (Figure 2B).

### The effect of discontinued vaccination on future outbreaks

If vaccination efforts are discontinued, the proportion of susceptible individuals within the MSM population is expected to increase gradually over time due to demographic turnover, as young individuals entering the at-risk group are likely unvaccinated. We modelled the impact of this rising susceptibility, while assuming a stable MSM population size and variable turnover rates ranging from 1% to 5% per year based on scenario 2 (range of seroprevalence levels between 35% and 55%, reduction of sexual contacts by MSM in response to a future outbreak). Our projections indicate that the median size of a potential outbreak in 10 years could vary between 254 cases (IQR: 152–363, 1% turnover rate per year) and 725 cases (IQR: 523–912, 5% turnover rate per year) if vaccination of at-risk individuals is discontinued (Figure 2E).

## Discussion

We showed a seroprevalence for VACV-specific antibodies of 45.4% and 47.1% among MSM visiting CSH in Amsterdam and Rotterdam, the Netherlands, respectively. Using mathematical modelling, we showed that vaccination efforts had minimal influence on the interruption of the Dutch 2022-23 outbreak, but reduce the likelihood and size of future mpox outbreaks. However, to achieve complete prevention, it is essential to keep time-to-diagnosis short, similar to the later stages of the 2022–23 Dutch outbreak. This requires maintained diagnostic capacities and sustained disease awareness among healthcare professionals and the at-risk groups.

We used a stochastic approach to model outbreaks of mpox. A simulation of the Dutch outbreak demonstrated comparability of our model-generated data to real-world epidemiology data. Assuming no introduction of risk group vaccination during the outbreak, we observed only a slight elevation of cumulative case numbers, highlighting that subsidence of the outbreak likely occurred independent of vaccine-induced immunity. Notably, the peak of the outbreak in the Netherlands had already occurred before vaccination campaigns commenced. It was recently suggested that the decline of the outbreak in the Netherlands could have occurred due to infection-induced immunity and behavioural adaptations among highly sexually active MSM [36]. Modelling studies conducted in other non-endemic countries, including the US, the UK and Italy, also suggested behavioural adaptations, risk group awareness, and infection-induced immunity as likely causes of the decline of the outbreak [37-40].

Like any modelling study, the reliability of our findings depends on the underlying assumptions of the model and the data used. A strength of our study is that we conducted simulations using seroprevalence levels measured among MSM in the Netherlands. We acknowledge that seroprevalence does not necessarily indicate sterilising immunity, therefore, our model does not assume that seropositive individuals are fully protected against infection. Additionally, our analysis benefits from incorporating published data on the serial interval [30] and vaccine effectiveness [7-9,16] that became available during the outbreak. The practical applicability of mathematical modelling is enhanced by the integration of real-world population immunity data, thereby supporting the formulation of targeted public health responses, including effective vaccination strategies. Furthermore, we included the intended reduction of sexual risk practices among MSM in response to the 2022–23 outbreak [27,41]. However, it is important to recognise that different sexual activity groups have varying contact rates and that the probability of mpox transmission per sexual encounter is unknown. The limited data led us to choose a non-assortative mixing assumption in our model. Predominant transmission within closely related networks of sexually active MSM may affect the outcome of this model; we acknowledge that our assumptions may result in an overestimation of future outbreak risks. Another complex behavioural aspect to model is self-isolation. In a survey conducted among Dutch MSM during the recent outbreak, 44% of respondents indicated a strong inclination towards self-isolation following an mpox diagnosis [42]. This, along with factors such as symptom recognition and healthcare-seeking behaviour, was incorporated by calibrating the model to the actual Dutch outbreak data and including ranges rather than fixed estimates. These combined factors highlight the intricacies of the actual transmission dynamics and potential biases in the model, urging a careful interpretation of its projections.

Serum samples used for our cross-sectional analysis were collected in September 2022, a period characterised by a rapid decline in the incidence of mpox cases. The vaccination campaign in the Netherlands commenced in July 2022, targeting high risk groups [4]. Thus, the timing of this serosurvey may have been too early to capture all seroconversions due to the relatively recent administration of vaccinations. To account for the possibility of low antibody titres shortly after vaccination, cut-off values in the VACV IgG ELISA were carefully defined while ensuring high sensitivity and specificity. Considering the potentially higher seroprevalence rates in subsequent months, a wider range of seroprevalence levels (up to 55%) was included in the sensitivity analysis of the stochastic model. Even at the upper end of this seroprevalence range, levels of immunity were insufficient to completely prevent future outbreaks in our model. In addition, vaccination- or infection-induced immunity against mpox is expected to further decline over time, which is indicated by the occurrence of breakthrough infections [10,18,19].

To prevent future mpox outbreaks, public health policy should take several factors into consideration. Firstly, population immunity against mpox can decline due to demographic changes within the risk group. Assuming turnover rates between 1% and 5%, we simulated median outbreak sizes between 254 cases (IQR: 152–363) and 725 cases (IQR: 523–912) 10 years from now, respectively, up from 179 cases (IQR: 108–265) without population turnover. Secondly, little is known about the longevity of immunity against mpox induced by third-generation smallpox vaccines or previous infection. Accordingly, our simulation of population turnover does not take waning of immune responses into account. Recent studies reported a decline in IgG levels 3 months and 1 year post MVA-BN vaccination, predominantly in individuals who had not received childhood smallpox vaccination [43,44]. As a consequence, our model-generated outbreak sizes 10 years from now likely underestimate total case numbers, although the correlate of protection against mpox disease is still unknown. In order to assess these changes, longitudinal or repeated cross-sectional studies are needed to monitor immunity levels in at-risk populations. Such activities can identify gaps in vaccination coverage across different age groups, enabling focused efforts on younger, previously unvaccinated individuals. Alternatively, it could be necessary to offer booster vaccinations to those previously vaccinated [45]. Thirdly, without ongoing outreach and education efforts, there is a concern that MSM and health professionals may become less aware of the symptoms of mpox over time, potentially leading to increased transmission of the disease.

It is important to note that the outcomes of this modelling study are not directly applicable to the situation in other countries, particularly endemic countries on the African continent. Our model takes the infection- and vaccination-based seroprevalence levels in the Netherlands, a non-endemic country, after the 2022–23 outbreak of MPXV clade IIb into account and was calibrated to parameters derived from that outbreak. This is markedly different from the endemic situation on the African continent where MPXV is enzootic and different taxonomical clades of the virus circulate among humans via different transmission routes. Monkeypox virus clade I (previously known as the Congo Basin clade), which has primarily been documented in the Democratic Republic of the Congo, is associated with greater disease severity and an increased mortality compared with MPXV clade II [46,47]. Moreover, human-to-human transmission of MPXV clade I has been described to occur through close contacts, however, recently was also linked to transmission via sexual contact (March 2023) [48]. Taken together, this results in distinctly different baseline scenarios, hampering the direct translation of our findings to the situation in endemic countries. Considering the recently reported increase of mpox cases in the Democratic Republic of the Congo [49], it is nevertheless critical to underline the need to investigate targeted approaches, which take into account the unique challenges of each setting.

## Conclusion

Our study underlines the importance of maintaining mpox-specific immunity in the at-risk population, alongside diagnostic capacities, continuous surveillance and sustained awareness among healthcare professionals and those at risk. These measures are vital for promptly identifying cases and implementing necessary control strategies. In addition, studies are needed to optimise vaccination of persons at risk of spillover infections in endemic countries. Future research should focus on understanding the longevity of vaccine-induced protection, contributing to a more comprehensive understanding of mpox epidemiology and facilitating targeted preventive measures.

## Supplementary Data

459000 Supplementary Material
