# Supplementary material for: Scenarios of future mpox outbreaks among men who have sex with men: a modelling study based on cross-sectional seroprevalence data from the Netherlands, 2022
Source: Euro Surveill. 2024 Apr 25;29(17):2300532. doi: 10.2807/1560-7917.ES.2024.29.17.2300532 (PMC11063670; doi:10.2807/1560-7917.ES.2024.29.17.2300532)
Supplement: Supplementary Material [file 23-00532_VAN_KESSEL_Supplement.pdf]

This supplementary material is hosted by *Eurosurveillance* as supporting information alongside the article ‘Scenarios of future mpox outbreaks among men who have sex with men: a modelling study based on cross-sectional seroprevalence data from the Netherlands, 2022’, on behalf of the authors, who remain responsible for the accuracy and appropriateness of the content. The same standards for ethics, copyright, attributions and permissions as for the article apply. Supplements are not edited by *Eurosurveillance* and the journal is not responsible for the maintenance of any links or email addresses provided therein.

Supplementary Figures

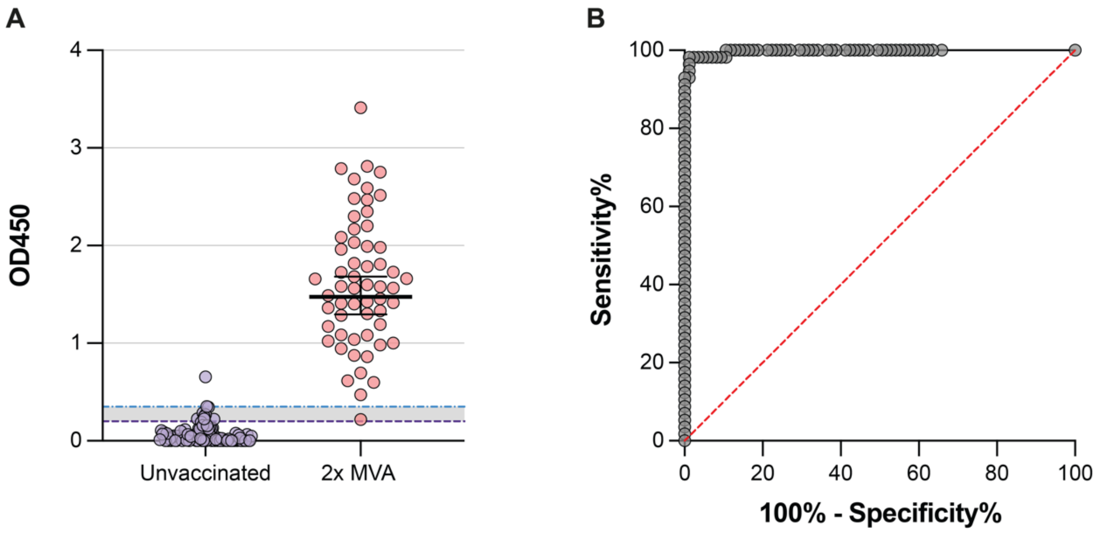

Supplementary Figure S1. Validation of the VACV-specific IgG screening ELISA.
(**A**) Distribution of VACV-specific IgG ELISA OD_{450} values in a validation set of 85 sera from orthopoxvirus-naive individuals (expected negative; purple), and a set of 57 sera from double-dose MVA-BN-vaccinated individuals collected 28 days after the second dose (expected positive; red). Samples were interpreted as negative with an $OD_{450}<0.2$, as borderline-positive with an OD_{450} between 0.2 and 0.35 (grey-shaded area), and as positive with an OD_{450} above 0.35. (**B**) An ROC curve was calculated based on the OD_{450} values of the validation set described above (area under the ROC curve = 0.9975 [95% CI 0.9932 – 1.000], $p < 0.0001$).

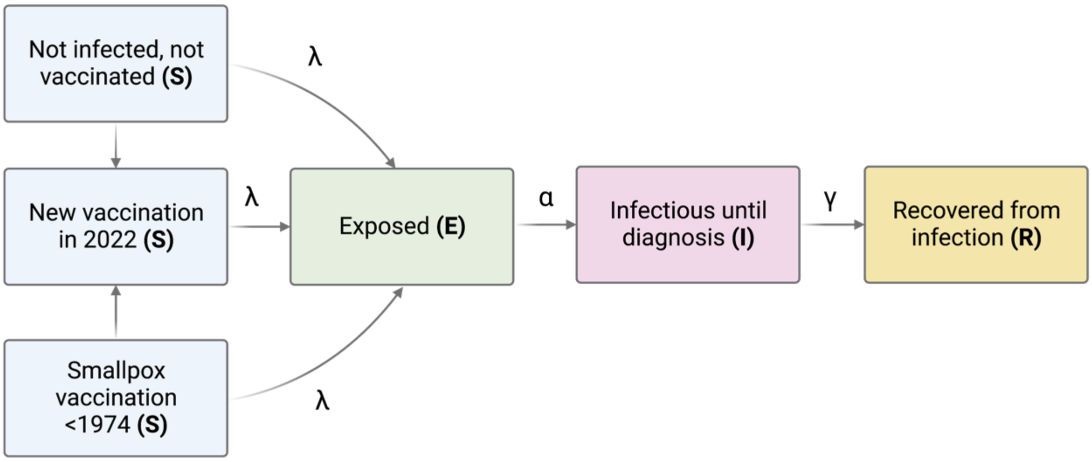

Supplementary Figure S2. Schematic representation of the mpox transmission model.
The Susceptible (S) – Exposed (E) – Infectious (I) – Removed (R) model includes three groups of susceptibles (S): naive (not previously infected, unvaccinated), vaccinated with a third-generation smallpox vaccine in 2022, and historically vaccinated before 1974. Susceptibles (S) become exposed to the virus through contact with an infectious individual at a rate of λ. Exposed individuals (E) become infectious (I) at a rate of α, and are removed from the model at a rate of γ, after which they are no longer infectious (R). The arrows in the diagram represent the movement of individuals between compartments. Parameters are further defined in **Supplementary Table 1**.

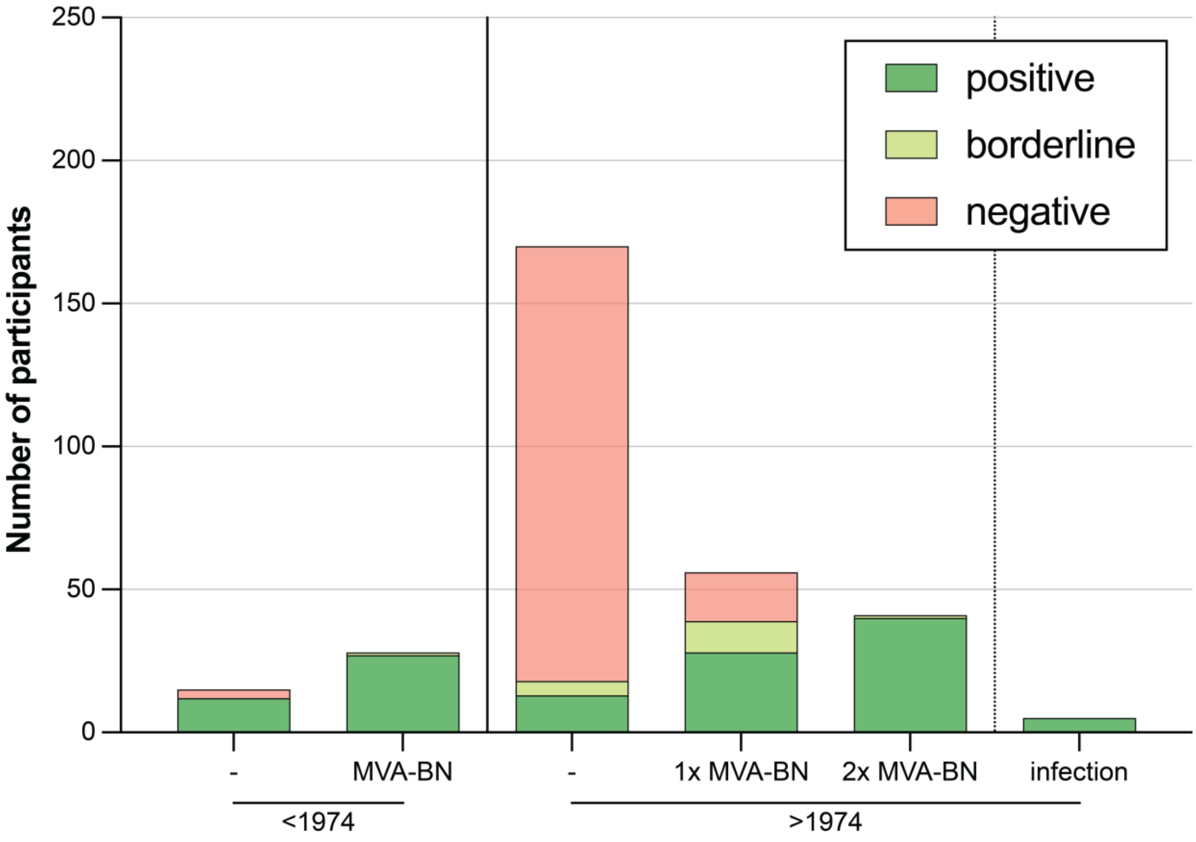

Supplementary Figure S3. Seroprevalence of VACV-specific antibodies among men who have sex with men (MSM) in Rotterdam.

Detection of VACV-specific IgG in n = 315 serum samples from MSM visiting the Centre for Sexual Health in Rotterdam using an in-house screening ELISA stratified by vaccination and infection status. Samples were considered positive with an OD_{450}>0.35 (green), borderline-positive with an OD_{450} between 0.35 and 0.2 (yellow), and negative with an OD_{450}<0.2 (red). Individuals born before 1974 (cessation of smallpox vaccination for the general population in the Netherlands) were inferred to have received childhood smallpox vaccination. VACV, vaccinia virus; MVA-BN, modified vaccinia virus Ankara – Bavarian Nordic.

Supplementary Tables

Supplementary Table S1. Parameters and corresponding values used in the model.

Overview of the definitions of parameters in equations 1-6 (see Supplementary Methods), and the corresponding ranges and references.

Parameter	Description	Range	Reference
S	MSM at risk of mpox in the Netherlands	45,000-60,000	Assumption
Vnew	Newly vaccinated	At end of base case scenario 14,000-22,000	Data
Vhist	Historically vaccinated against smallpox	10%-20%	Data
E	Exposed, but not infectious	0 at start	Assumption
I	Infectious	1-10 at start of outbreak	Assumption
R	Recovered from mpox	0 at start	Assumption
λ	Transmissibility of mpox	0.5-1	Calibrated
Vaccinated	Number of MSM vaccinated during outbreak	240-360 per day	Calibrated
VEnew	Vaccine effectiveness MVA-BN	78% (95% CI 54%-89%)	[1-4]
VEhist	Vaccine effectiveness historical smallpox vaccination	85% (range 75%-95%)	[5]
N	All MSM at risk of mpox		
$pVhist_{(t=0)}$	Proportion historically vaccinated before 1974 at start of outbreak	10-20%	Data[6]
α	Serial time	8.0 (95% CI 6.5-9.9 days)	[7]
γ	Time from symptom onset to diagnosis or virus clearance	1-21 days (period 1)<br>4-7 days (period 2)	Calibrated

Supplementary Table S2. Cumulative outbreak size in modelled scenarios of future mpox outbreaks among men who have sex with men (MSM) in the Netherlands.
Numerical overview of the cumulative outbreak sizes for each scenario modelled as a supplement for Figures 2C and 2D.

Scenario	Description	Cumulative outbreak size (IQR)
Dutch 2022-23 outbreak (modelled)	Simulation of the 2022–23 Dutch mpox outbreak using our model	1,325 (1,262–1,419)
Scenario 1	Simulation assuming that no vaccination campaign had occurred in 2022 and only individuals with prior infections or childhood vaccinations would be (partially) protected against infection	1,105 (1,000–1,206)
Scenario 2	A partially vaccinated population (seroprevalence 35–55%) with a reduction of sexual partners within the risk group comparable to the original outbreak	179 (108–265)
Scenario 3	Same as scenario 2 but without a reduction of sexual contacts	344 (167–526)
Scenario 4	A partially vaccinated population (seroprevalence 35–55%) with a decreased time-to-diagnosis comparable to the end of the outbreak	2 (0–6)
Scenario 5	Same as scenario 4 but with a reduction of sexual contacts	2 (0–6)

Supplementary Methods

Equations used in the stochastic model

The stochastic model based on the Gillespie algorithm[8] can be mathematically described using the following equations:

(1) $S_{(t+1)} = S_{(t)} - \frac{\lambda * S(t) * I}{N} - Vaccinated_{(t)}$

(2) $Vnew_{(t+1)} = Vnew_{(t)} - \frac{\lambda * (1 - VEnew) * Vnew(t) * I}{N}$

(3) $Vhist_{(t+1)} = Vhist_{(t)} - \frac{\lambda * (1 - VEhist) * Vhist(t) * I}{N} - Vaccinated_{(t)} * pVhist_{(t=0)}$

(4) $E_{(t+1)} = E_{(t)} + \frac{\lambda * S(t) * I}{N} + \frac{\lambda * (1 - VEnew) * Vnew(t) * I}{N} + \frac{\lambda * (1 - VEhist) * Vhist(t) * I}{N} - E_{(t)} * \alpha$

(5) $I_{(t+1)} = I_{(t)} + E_{(t)} * \alpha - I_{(t)} * \gamma$

(6) $R_{(t+1)} = R_{(t)} + I_{(t)} * \gamma$

Calibration of the model

The model was run 1,000,000 times in MATLAB. A total of 439 simulations were selected, which matched the 2022 outbreak including:

- The cumulative number of mpox cases during the 2022-23 outbreak (number of 1,200-1,800).
- A deviation of at most 50% in the number of newly reported cases during the first four months of the outbreak (95, 454, 476 and 170 in the first, second, third, and fourth month, respectively)
- The number of newly vaccinated individuals (range 14,000-22,000)
- The seroprevalence of mpox (range 35%-55%)

Analysis of the model

For each simulation a unique seeding number was selected, which was subsequently re-used in the analysis of the model to ensure that the same random numbers were chosen in the calibration and in the analysis. In the analysis, we compared five different scenarios:

1. Simulation assuming that no vaccination campaign had occurred in 2022 and only individuals with prior infections or childhood vaccinations would be (partially) protected against infection
2. A partially vaccinated population (seroprevalence 35–55%) with a reduction of sexual partners within the risk group comparable to the original outbreak
3. Same as scenario 2 but without a reduction of sexual contacts
4. A partially vaccinated population (seroprevalence 35–55%) with a decreased time-to-diagnosis comparable to the end of the outbreak
5. Same as scenario 4 but with a reduction of sexual contacts

Sensitivity analysis

In a sensitivity analysis, we investigated the impact of the seroprevalence (35%-<45% vs 45%-<55%) or a different effectiveness of the MVA-BN vaccine (<65%, 65%-<75%, 75%-<85% and >85%) on the cumulative number of mpox diagnoses during a new potential outbreak (**Figure 2B**).

Supplementary References

1. Deputy NP, Deckert J, Chard AN, et al. Vaccine Effectiveness of JYNNEOS against Mpox Disease in the United States. *N Engl J Med* 2023.
2. Payne AB, Ray LC, Cole MM, et al. Reduced Risk for Mpox After Receipt of 1 or 2 Doses of JYNNEOS Vaccine Compared with Risk Among Unvaccinated Persons - 43 U.S. Jurisdictions, July 31-October 1, 2022. *MMWR Morb Mortal Wkly Rep* 2022; **71**(49): 1560-4.
3. Wolff Sagy Y, Zucker R, Hammerman A, et al. Real-world effectiveness of a single dose of mpox vaccine in males. *Nat Med* 2023; **29**(3): 748-52.
4. Xu M, Liu C, Du Z, Bai Y, Wang Z, Gao C. Real-world effectiveness of mpox (monkeypox) vaccines: a systematic review. *J Travel Med* 2023.
5. Jezek Z, Grab B, Szczeniowski MV, Paluku KM, Mutombo M. Human monkeypox: secondary attack rates. *Bull World Health Organ* 1988; **66**(4): 465-70.
6. Zaeck LM, Lamers MM, Verstrepen BE, et al. Low levels of monkeypox virus-neutralizing antibodies after MVA-BN vaccination in healthy individuals. *Nat Med* 2023; **29**(1): 270-8.
7. Ward T, Christie R, Paton RS, Cumming F, Overton CE. Transmission dynamics of monkeypox in the United Kingdom: contact tracing study. *BMJ* 2022; **379**: e073153.
8. Keeling MJR, P. Modeling infectious diseases in humans and animals. *Princeton: Princeton University Press* 2008.
